# From Likes to Lifestyle: Predicting the Impact of Following Fitness Influencers on the Adoption of Healthy Habits in Saudi Arabia

**DOI:** 10.7759/cureus.64710

**Published:** 2024-07-17

**Authors:** Najim Z Alshahrani, Abdulrahman M Albeshry, Mohamed Terra, Mohamed Baklola, Mohammed Yahya Al alshaei, Ali Yahya Al alshahi

**Affiliations:** 1 Department of Family and Community Medicine, Faculty of Medicine, University of Jeddah, Jeddah, SAU; 2 Faculty of Medicine, Mansoura University, Mansoura, EGY; 3 Public Health, Mansoura University, Mansoura, EGY; 4 College of Medicine, Najran University, Najran, SAU

**Keywords:** saudi arabia, eating habits, nutrition, public health, health behaviors, fitness influencers, social media

## Abstract

Background: The increasing popularity of fitness influencers on social media has the potential to significantly impact public health by promoting healthy behaviors. Understanding how these influencers affect the adoption of healthy habits among Saudi residents can provide insights into effective public health strategies.

Objective: This study aims to quantitatively assess the influence of following fitness influencers on social media on adopting healthy behaviors among Saudi residents, focusing on socio-demographic factors, characteristics of influencers, and participants’ perceptions.

Methods: A descriptive, cross-sectional study with an analytical component was carried out from March 25, 2023, to August 15, 2023. The study included Saudi residents aged 18 and older who actively follow fitness influencers. Data was gathered through an online questionnaire and analyzed using descriptive statistics, Pearson’s chi-square test, and logistic regression analyses to identify factors associated with positive health outcomes.

Results: The study included 561 participants, revealing that marital status and residential region significantly influenced the adoption of healthy habits, with married participants and those from the Southern and Central regions more likely to report positive outcomes. Notably, engagement in physical activity more than three times a week and adherence to a healthy diet were strongly associated with positive health outcomes (p<0.05). Analysis of influencer characteristics showed that following fewer than 10 influencers and being unaware of their follower count were predictors of positive results with p<0.05. Specifically, 76.6% of participants experienced positive results after following health advice from social media, highlighting the impactful yet complex role of influencers in shaping health behaviors. Logistic regression analyses underscored the significance of socio-demographic factors, influencer characteristics, and participant perceptions in predicting the likelihood of experiencing positive health outcomes.

Conclusion: Following fitness influencers on social media can positively impact the adoption of healthy habits, moderated by socio-demographic factors and influencer characteristics.

## Introduction

In the digital era, social media has revolutionized how information is disseminated and consumed, particularly concerning health and fitness [1]. Globally, fitness influencers on platforms like Instagram, Twitter, and YouTube have gained substantial followings, leveraging their reach to impact public health behaviors and attitudes [2]. This phenomenon has not bypassed Saudi Arabia, a country experiencing a digital transformation alongside a growing health and fitness awareness [1].

The rise of fitness influencers can be traced back to the early 2000s, with the advent of social media platforms [3]. These individuals, often without formal health education, command significant influence over their audience’s lifestyle choices by sharing workout routines, dietary advice, and personal health journeys [3]. Their impact is augmented by the visual and interactive nature of social media, which provides a persuasive platform for health promotion.

In Saudi Arabia, this trend coincides with a critical public health context. The country faces unique health challenges, predominantly lifestyle-related diseases such as obesity, diabetes, and cardiovascular disorders [4]. The Saudi Ministry of Health has acknowledged these issues and actively promotes healthier lifestyles. However, traditional health promotion methods face limitations in reach and engagement, particularly among the younger population [5]. In contrast, social media offers an accessible and engaging avenue to disseminate health information and influence behavior, especially given the high internet penetration and social media usage in the country [6-9].

A study conducted in Saudi Arabia in 2024 explored the specific role of YouTube influencers, revealing that these influencers significantly affect both positive and negative health behaviors, particularly in terms of diet and smoking cessation [10]. The study highlights the varied impacts depending on the type of influencer, with healthcare influencers being the most positively received. Additionally, another study in 2024 illustrates how fashion influencers also partake in shaping health behaviors, albeit with mixed outcomes. Notably, this research identified a relationship between the educational level of followers and their susceptibility to negative health influences from fashion influencers, emphasizing the need for critical content evaluation [11].

The role of influencers in shaping public health behaviors has been a subject of increasing research interest [12]. Studies have shown that social media can effectively promote physical activity, healthy eating, and mental well-being. Influencers, with their relatable personas and persuasive narratives, often serve as catalysts for behavior change [13]. They can demystify health information, making it more approachable and applicable to everyday life. However, the credibility and accuracy of the information they provide have been areas of concern, with potential risks of misinformation [14].

In Saudi Arabia, the convergence of health, social media, and cultural dynamics creates a distinct environment [15]. The nation’s youthful demographic, widespread smartphone use, and shifting social norms provide a rich setting for digital health promotion. Fitness influencers, predominantly Saudis, engage the local audience by integrating health advice with culturally relevant content. They offer not only health guidance but also embody a lifestyle aspiration within the Saudi socio-cultural milieu.

This study seeks to examine the impact of following fitness influencers on the adoption of healthy habits among Saudi individuals. It aims to evaluate socio-demographic and influencer-related factors associated with positive health outcomes, assess Saudi perceptions of the credibility and influence of fitness influencers on their health decisions, and use binary logistic regression analysis to measure these effects. This research is especially pertinent given Saudi Arabia’s ongoing digital transformation and growing focus on public health and wellness, aiming to offer a comprehensive empirical insight into this contemporary health communication phenomenon.

## Materials and methods

Survey design and participants

This study employed a descriptive, cross-sectional design with an analytic component, targeting the general population of Saudi Arabia between August 25, 2023, and January 15, 2024. Participants were required to be Saudi residents aged 18 years or older and active followers of fitness influencers on social media. Individuals, unwilling to participate, were excluded from the study.

Sample size and data collection approach

The minimum required sample size for the study was calculated using the Raosoft sample size calculator with the following parameters: a population proportion of 50%, a margin of error of 5%, and a confidence interval (CI) of 95%. Using the formula: n = (Z^2^ * p * (1 - p)) / e^2^. Where Z is 1.96 (corresponding to a 95% confidence level), p is 0.5 (the assumed population proportion), and e is 0.05 (the margin of error), the minimum required sample size was determined to be 384 participants. To ensure the robustness of the results and account for potential dropouts and non-responses, we targeted a minimum of 500 participants for this study. The survey was completed by 561 participants.

Convenient sampling was employed to recruit study participants due to its practicality and efficiency in reaching a large number of individuals quickly. An Arabic-structured, validated questionnaire was developed and hosted on Google Forms. The survey link was then distributed through various social media platforms, including WhatsApp, Facebook, and Twitter, to ensure a diverse group of Saudi residents could be reached. Participants were required to provide informed consent before proceeding with the questionnaire, acknowledging a statement that explained the voluntary nature of their participation and assured them of confidentiality. Individuals who clicked the survey link and agreed to the informed consent statement were directed to complete the questionnaire, and reminders were included to encourage participants to answer all questions to ensure their responses were valid. Participants who did not follow fitness influencers or were unwilling to participate were excluded from the study.

In this study, “Followers” are operationally defined as individuals who actively follow fitness influencers on social media platforms. This includes not only those who officially subscribe or follow the influencers’ accounts but also those who regularly view the videos, reels, and posts of these influencers and make lifestyle changes based on the content. Specific questions were included in the questionnaire to determine participants’ engagement levels and whether they considered themselves followers of fitness influencers.

Study tools

The study utilized a structured questionnaire that was developed specifically for this research through an extensive literature review. The questionnaire was divided into four sections, each designed to gather comprehensive data relevant to the study’s objectives. The first section of the questionnaire collected socio-demographic data, including information on participants’ location, gender, age, education level, employment status, and monthly income. The second section included questions about participants’ current health status, frequency of physical activity, dietary habits, body mass index (BMI), and smoking status. The third section examined the attributes of fitness influencers followed by the participants. Questions in this section addressed the number and type of influencers followed, the gender of the influencers, the nature of the content provided (e.g., fitness tips, dietary advice), and the perceived credibility of these influencers. The fourth section explored participants’ perceptions and the impacts of fitness influencers on their health attitudes and behaviors. This included questions about participants’ trust in the influencers’ advice, the extent to which they believed influencers adhered to their own health advice, and whether participants had experienced any positive changes in their health behaviors as a result of following these influencers.

To ensure the questionnaire’s clarity and reliability, it was pre-tested with 30 Saudi participants. The feedback from this pilot study led to minor adjustments for clarity. Additionally, the questionnaire was evaluated by four public health and sociology professors, who assessed it for content validity and reliability. The pilot study results indicated moderate to good internal consistency, with a Cronbach’s α reliability coefficient of 0.73, demonstrating the reliability of the instrument.

Ethics approval

This study adhered to the principles outlined in the Declaration of Helsinki and received approval from the Institutional Review Board of the Research Ethics Committee at King Faisal University (protocol code: KFU-REC-2022-DEC-EA000418; date of approval: 01/10/2022). Informed consent was obtained from all participants after they were informed of the study’s objectives. Participation was entirely voluntary, with no remuneration provided.

Statistical analysis

Data were initially coded and organized in Microsoft Excel for quality control before being imported into SPSS (version 25.0) for analysis. Descriptive statistics, including percentages, frequencies, means, and standard deviations, were used to summarize the participants’ demographic characteristics and their responses. Pearson’s chi-square test was applied to compare categorical variables between groups. Additionally, logistic regression was performed to investigate the relationship between following fitness influencers and achieving positive health outcomes among participants. Both univariate and multivariate logistic regression analyses were conducted. The univariate analysis identified individual factors significantly associated with positive health outcomes, while the multivariate analysis adjusted for potential confounders to provide a comprehensive understanding of independent predictors of health behavior changes. Odds ratios (ORs) with 95% CIs were calculated to assess the strength and direction of associations. A p-value of less than 0.05 was deemed statistically significant in all analyses.

## Results

Socio-demographic and health-related characteristics

Our study analyzed participants’ socio-demographic and health-related characteristics and their correlation with positive outcomes from adopting fitness and health habits. The average age of participants who experienced positive results was 30.4 years, compared to 29.3 years for those who did not; this difference was not statistically significant (p=0.09). Gender distribution showed no significant difference in outcomes, with 62 males (24.4%) and 69 females (22.5%) reporting positive results (p=0.61). Marital status emerged as a significant factor, with single individuals (N=57, 19.7%) less likely to report positive outcomes compared to married participants (N=74, 27.3%, p<0.05). The residential region was significantly associated with positive outcomes. Participants from the Southern region (N=21, 16.7%) and the Central region (N=55, 46.6%) reported higher positive outcomes compared to other regions (p<0.05). Educational level and monthly family income showed no significant correlation with adopting healthy habits (p=0.47 and p=0.51, respectively). Similarly, BMI categories and smoking status did not significantly influence the adoption of healthy habits (p=0.54 and p=0.16, respectively). Notably, the frequency of physical activity and adherence to a healthy diet were significantly associated with positive outcomes. Participants engaging in physical activity more than three times a week (N=6, 5.6%) and those following a healthy diet (N=35, 14.1%) were more likely to report positive health outcomes (p<0.05 for both), as shown in Table 1.

**Table 1:** Socio-demographic and Health-Related Characteristics of Participants, Stratified by Positive Outcomes from Adopting Fitness and Health Habits

Variable		Experienced Positive Results (N=131)	Did Not Experience Positive Results (N=430)	Total (N=561)	P-value
Age (Mean ±SD)		30.4 (12.1)	29.3 (9.8)		0.09
Gender	Male	62	192	254	0.6
	Female	69	238	307	
Marital status	Single	57	233	290	<0.05
	Married	74	197	271	
Residence	Southern region	21	105	126	<0.05
	Eastern region	19	92	111	
	Northern region	14	61	75	
	Western region	22	109	131	
	Central region	55	63	118	
Educational level	Secondary School	36	116	152	0.47
	Bachelor’s degree	87	273	360	
	Post-graduate’s degree	8	41	49	
Field of work	Medical	11	86	97	<0.05
	Non-medical	45	123	168	
	Student	29	160	189	
	Not employed	46	61	107	
Monthly family income (*Saudi Riyal*)	Less than 3,000	18	67	85	0.51
	3,001- 10,000	18	80	98	
	10,001-20,000	69	202	271	
	More than 20,000	26	81	107	
Body mass index (BMI)	Underweight	8	61	69	0.54
	Normal‎weight	62	207	269	
	Overweight	40	112	152	
	‎Obese	21	50	71	
Current Smoker	Yes	40	105	145	0.16
	No	91	325	416	
Frequency of physical activity/ Week	No physical activity	59	129	188	<0.05
	Once	43	98	141	
	Two to three times	23	101	124	
	More than three times	6	102	108	
Following healthy diet	Yes	35	214	249	<0.05
	No	96	216	312	
How do you describe your health status in general?	Bad	20	49	69	0.13
	Good	59	236	295	
	Excellent	52	145	197	

Influencer characteristics and their impact

Table 2 shows the characteristics of fitness influencers followed by participants who showed varying impacts. The gender of influencers did not significantly affect the outcomes (p=0.057). However, the number of influencers followed showed a trend toward significance, with those following fewer than 10 influencers more likely to report positive results (p=0.056). Participants unaware of their influencers’ follower count were more likely to experience positive outcomes (p<0.05). The nature of health tips provided by influencers also significantly influenced outcomes, with participants reporting positive results more likely to follow influencers who offer health tips (p<0.05).

**Table 2:** Characteristics of Fitness Influencers Followed, Stratified by Positive Outcomes from Adopting Fitness and Health Habits

Characteristics/Variables		Experienced Positive Results (N=131)	Did Not Experience Positive Results (N=430)	Total (N=561)	P-value
Gender of influencers followed	Female	9	64	73	0.057
	Male	31	96	127	
	Both	91	270	361	
Average number of influencers followed	<10	51	172	223	0.056
	10-50	52	156	208	
	50-100	24	59	83	
	100+	4	43	47	
Approximate average number of followers of influencers followed	Don’t know	74	167	241	<0.05
	<10 k	1	23	24	
	10 k-100 k	14	89	103	
	100 k-1 m	25	104	129	
	1 m+	17	47	64	
Do the influencers on social media whom you specifically choose to follow provide health tips on their platforms?	No	33	33	66	<0.05
	Some of them	89	332	421	
	Neutral	9	19	28	
	Yes, all of them	0	46	46	
Do the influencers on social media whom you follow promote a daily healthy lifestyle?	No	7	18	25	<0.05
	Some of them	90	329	419	
	Neutral	31	48	79	
	Yes, all of them	3	35	38	

Perceptions and impact of influencers on health attitudes and behaviors

The majority of participants (199, 77.1%) believed that influencers only sometimes adhere to the health advice they provide. Trust in health advice from non-healthcare professional influencers was predominantly conditional, with 483 (86.1%) participants sometimes trusting such advice. A significant portion (404 participants, 72.0%) felt that the promotion of healthy products by influencers was motivated by monetary gains. Notably, 430 (76.6%) participants reported experiencing positive results after following health advice from social media. This finding highlights the substantial impact of influencers on health behaviors. Participants' views on influencers' commitment to healthy habits and their representation of a healthy lifestyle were varied, with only a minority (240 participants, 42.8%, and 59 participants, 10.5%, respectively) perceiving these aspects positively (Table 3).

**Table 3:** Perception and Impact of Influencers on Health Attitudes and Behaviors

Variable		Frequency	Percentage
Do social media influencers adhere to the health advice they provide to the public?	Always	47	18.2
	Sometimes	199	77.1
	Never	12	4.7
Do you trust health advice given by influencers on social media who are not in the healthcare field?	Always	24	4.3
	Sometimes	483	86.1
	Never	54	9.6
Do you think that the promotion of healthy products by social media influencers is solely for monetary purposes?	Always	124	22.1
	Sometimes	404	72.0
	Never	33	5.9
Have you ever experienced positive results after following the health advice and habits you learned on social media?	No	131	23.4
	Yes	430	76.6
Have you noticed that influencers who are committed to proper healthy habits tend to have good health?	Some of them	305	54.4
	No	16	2.9
	Yes	240	42.8
Do you agree that social media influencers represent an accurate image of a healthy lifestyle?	Some of them	421	75.0
	No	81	14.4
	Yes	59	10.5
Overall, to what extent are you satisfied with the changes that have occurred in your lifestyle due to the influence of celebrities on social media?	Completely satisfied	108	19.3
	Somewhat satisfied	379	67.6
	Not satisfied	74	13.2

Binary logistic regression analysis: Socio-demographic factors

In the logistic regression analysis, marital status and field of work emerged as significant factors influencing the likelihood of experiencing positive results. Married individuals were less likely to experience positive results compared to singles in the univariate analysis, but this was not significant in the multivariate model (p<0.05 and p=0.54, respectively). Participants from non-medical fields and those not employed were significantly less likely to experience positive results (p<0.05 for both) (Table 4).

**Table 4:** Binary Logistic Regression Analysis of Experiencing Positive Results Regarding Sociodemographic * Italic and asterisk values indicate statistically significant (p <0.05); OR: odds ratio; CI: confidence interval; r: reference

Variables		Experiencing Positive Results*			
		Univariate Regression		Multivariate Regression	
		OR 95% (CI)	P-Value	OR 95% (CI)	P-Value
Demographic Factors					
Marital status	Single	1 (r)	<0.05	1 (r)	0.54
	Married	0.65 (0.44-0.96)		1.17 (0.69-2.01)	
Residence	Southern region	1 (r)		1 (r)	
	Eastern region	0.96 (0.49-1.91)	0.93	0.64 (0.3-1.3)	0.2
	Northern region	0.87 (0.41-1.84)	0.72	0.75 (0.34-1.6)	0.48
	Western region	0.99 (0.51-1.9)	0.98	0.79 (0.39-1.6)	0.5
	Central region	0.23 (0.13-0.41)	<0.05	0.22 (0.12-0.44)	<0.05
Field of work	Medical	1 (r)		1 (r)	
	Non-medical	0.35 (0.17-0.71)	<0.05	0.3 (0.14-0.65)	<0.05
	Student	0.7 (0.34-1.48)	0.36	0.67 (0.3-1.5)	0.31
	Not employed	0.17 (0.08-0.35)	<0.05	0.19 (0.08-0.4)	<0.05
Following healthy diet	No	1 (r)	<0.05	1 (r)	<0.05
	Yes	2.71 (1.76-4.18)		2.47 (1.56-3.9)	

Binary logistic regression analysis: Influencer characteristics and other factors

The analysis revealed that participants following influencers with fewer than 10k followers were significantly more likely to experience positive results (p<0.05). Additionally, those following influencers who provide health tips were more inclined to experience positive results (p<0.05), indicating the influencers’ role in helping people experience positive results, as shown in Table 5. Our binary logistic regression analysis focusing on “Other Factors” found that participants’ perceptions significantly influenced their health-related behaviors. Specifically, the belief in influencers’ adherence to their own health advice and the trust in health advice from non-healthcare influencers were pivotal. Participants who are skeptical of influencers’ adherence or those who distrust their health advice were more likely to experience positive results (p<0.05). This highlights the nuanced impact of influencer credibility and audience perceptions on health behavior, as shown in Table 5.

**Table 5:** Binary Logistic Regression Analysis of Experiencing Positive Results Regarding Influencer Characteristics *statistically significant (p <0.05); OR: odds ratio; CI: confidence interval; r: reference

Variables		Experiencing Positive Results*			
		Univariate Regression		Multivariate Regression	
		OR 95% (CI)	P-Value	OR 95% (CI)	P-Value
Influencer Characteristics					
Approximate average number of followers of influencers followed	Don’t know	1 (r)			
	<10k	0.3 (0.06-1.32)	0→ .1	0.23 (0.04-1.03)	<0.05
	10 k-100 k	0.24 (0.05-1.13)	0.07	0.2 (0.04-1)	<0.05
	100 k-1 m	0.34 (0.07-1.5)	0.15	0.26 (0.05-1.18)	0.08
	1 m+	1.15 (0.15-8.9)	0.89	1.05 (0.13-8.65)	0.95
Do the influencers on social media whom you specifically choose to follow provide health tips on their platforms?	No	1 (r)			
	Some of them	0.27 (0.15-0.46)	<0.05	0.27 (0.15-0.5)	<0.05
	Neutral	0.56 (0.24-1.29)	0.17	0.63 (0.26-1.58)	0.3
	Yes, all of them		0.99		0.99
Do the influencers on social media whom you follow promote a daily healthy lifestyle?	No	1 (r)		1 (r)	
	Some of them	0.7 (0.28-1.73)	0.4	1.2 (0.4-3.2)	0.72
	Neutral	0.42 (0.25-0.7)	<0.05	0.55 (0.3-0.99)	0.05
	Yes, all of them	3.19 (0.95-10.6)	0.058	1.02 (0.2-3.8)	0.96
Other Factors					
Do social media influencers adhere to the health advice they provide to the public?*	Always	1 (r)	-	1 (r)	-
	Sometimes	0.57 (0.18-1.8)	<0.05	1.35 (0.35-5.08)	0.65
	Never	1.7 (1.14-2.5)	<0.05	4.89 (0.83-28.85)	0.07
Do you trust health advice given by influencers on social media who are not in the healthcare field?*	Always	1 (r)	-	1 (r)	-
	Sometimes	1.65 (0.9-3.03)	0.1	1.59 (0.82-3.09)	0.1
	Never	11.5 (1.43-92.1)	<0.05	8.78 (1.05-73.4)	<0.05
Have you noticed that influencers who are committed to proper healthy habits tend to have good health?*	No	1 (r)	-		-
	Yes	0.39 (0.14-1.08)	0.07	3.67 (1.19-11.32)	<0.05
	Some of them	2.15 (1.4-3.31)	<0.05	1.75 (0.57-5.3)	0.32
Do you agree that social media influencers represent an accurate image of a healthy lifestyle?*	No	1 (r)	-		-
	Yes	3.36 (1.49-7.58)	<0.05	1.31 (0.53-3.28)	0.5
	Some of them	2.6 (1.57-4.29)	<0.05	2.12 (1.2-3.73)	-
Overall, to what extent are you satisfied with the changes that have occurred in your lifestyle due to the influence of celebrities on social media?*	Not satisfied	1 (r)	-		-

## Discussion

Our study embarked on a pioneering exploration into the dynamic interplay between following fitness influencers on social media and the subsequent adoption of healthy habits among the population of Saudi Arabia. The findings of our study shed light on the nuanced impact that following fitness influencers on social media has on the adoption of healthy habits among the Saudi Arabian population. This discussion delves into the implications of these findings, comparing them with existing literature, and suggesting areas for future research.

Our study’s results indicate that socio-demographic factors, such as marital status and residential region, significantly influence the adoption of healthy habits. Interestingly, while marital status showed a significant correlation in univariate analysis, its influence was not significant in the multivariate model. This suggests that other factors might moderate the relationship between marital status and health behavior changes. The significant association between residential regions and positive health outcomes, particularly in the Central and Southern regions, highlights geographical disparities that could be attributed to varying levels of access to fitness and health resources or differing cultural attitudes toward health and fitness across regions.

Comparatively, existing literature has also underscored the importance of socio-demographic factors in health behavior changes. For example, studies have shown that urban residents are often more exposed to health and fitness culture, possibly explaining the regional differences observed in our study [14,15]. Furthermore, the lack of significant differences based on gender contrasts with some studies suggesting women are more influenced by social media for health information [16], indicating a potential cultural shift or unique social dynamics within Saudi Arabia.

The characteristics of fitness influencers, including the number of influencers followed and the nature of the health tips provided were found to significantly impact participants’ health behaviors. This finding underscores the pivotal role influencers play in shaping health attitudes and behaviors, aligning with literature suggesting that influencers can effectively promote health information and behaviors among their followers [17,18]. Notably, the trend toward positive outcomes among participants following fewer influencers may reflect a more focused or quality-oriented approach to consuming health-related content, as opposed to the overwhelming or contradictory information that might come from following numerous sources [19].

The significant influence of health tips provided by influencers on positive health outcomes further emphasizes the importance of content quality over quantity [20]. It suggests that influencers who provide actionable, reliable health tips can have a more substantial impact on their followers’ health behaviors than those who do not. This is consistent with research indicating that the perceived credibility of health information on social media can significantly affect health behavior changes [21].

Our study also highlights the complex relationship between participants’ perceptions of influencers and the adoption of healthy habits. While a majority of participants were skeptical about influencers’ adherence to their health advice and questioned the motivations behind promoting health products, a significant portion reported experiencing positive health outcomes from following such advice. This paradox suggests that while trust in influencers may be conditional, the influence of social media on health behaviors is undeniable. The findings align with studies suggesting that even with skepticism toward the credibility of health information on social media, users can still find value in the content if it aligns with their health goals [22].

Comparing our findings with recent studies conducted in Saudi Arabia further contextualizes the impact of different types of influencers on health behaviors. A 2024 study on the role of YouTube influencers revealed that these influencers significantly affect both positive and negative health behaviors, particularly in terms of diet and smoking cessation [8]. This study highlighted that 71.7% of participants perceived influencers as encouraging healthy habits, yet 55.6% also believed they promoted unhealthy habits. Positive outcomes included dietary improvements (62.6%) and smoking cessation (20.5%), with healthcare influencers being the most positively received [8]. These results are consistent with our findings that influencer characteristics and the nature of health tips provided significantly impact health outcomes, underscoring the complex influence of social media on health behaviors.

Another 2024 study on the role of fashion influencers illustrated how these influencers also partake in shaping health behaviors, albeit with mixed outcomes [9]. The study found that postgraduate education was associated with negative health outcomes, emphasizing the need for critical content evaluation. Notably, individuals who followed fashion influencers offering health tips and advocating a healthy lifestyle had higher odds of negative outcomes if the influencers had fewer than 10,000 followers [9]. This finding aligns with our observation that following fewer influencers may lead to more positive health outcomes, highlighting the importance of influencer credibility and the quality of content consumed.

The findings from this study have several implications for public health strategies and social media policy. First, the significant role of influencers in promoting health behaviors points to the potential of leveraging influencer partnerships in public health campaigns [23]. However, it also underscores the need for regulatory frameworks to ensure the credibility and reliability of health information shared on social media platforms [3,24,25]. Second, the observed regional disparities in health outcomes suggest the importance of targeted health promotion efforts that consider geographical and cultural contexts.

Future research should explore the long-term impact of following fitness influencers on health behaviors and outcomes. Additionally, qualitative studies could provide deeper insights into the motivations behind following fitness influencers and how individuals discern and apply health information from social media. Investigating the role of influencers in other health-related areas, such as mental health or chronic disease management, could also offer valuable insights.

Strengths and limitations of this study

This study boasts several strengths, including its comprehensive approach to examining the impact of fitness influencers on health behaviors in a specific cultural context. Focusing on Saudi Arabia provides valuable insights into how digital health promotion operates within unique socio-cultural dynamics. The use of a cross-sectional design with a sizable sample size and the incorporation of both demographic factors and personal perceptions about influencers enhance the study’s robustness.

Methodologically, the application of various statistical analyses, including logistic regression, offers a thorough understanding of the factors influencing health behaviors. However, the study is not without limitations. Its reliance on convenience sampling may limit the generalizability of the findings, as participants are self-selected and may not represent the broader Saudi population.

The cross-sectional nature of the study only captures a snapshot in time, preventing the establishment of causal relationships. Additionally, the self-reported data could be subject to bias, as participants may provide socially desirable responses or misremember their behaviors. The focus on followers of fitness influencers also excludes the perspectives of those who do not engage with such digital content, potentially overlooking other significant factors influencing health behaviors in the general population.

Finally, the study did not extensively explore the impact of the credibility and expertise of influencers, which could be a critical factor in determining the effectiveness of their health advice.

## Conclusions

Our study sheds light on the intricate dynamics of how fitness influencers on social media can shape healthy habits among Saudi residents. We found that socio-demographic factors and the quality of content from fitness influencers play pivotal roles in influencing health behavior changes. Despite some skepticism regarding the authenticity and motivations of these influencers, many participants reported significant positive health outcomes from following social media advice. This highlights a fascinating interplay between trust and influence, suggesting that even amidst doubts, credible and engaging health content can drive meaningful behavioral changes. These findings emphasize the critical need for trustworthy and actionable health information. They also reveal a valuable opportunity for public health strategies to harness the power of social media influencers to promote healthy behaviors effectively. By focusing on the credibility of the influencers and the quality of their content, public health campaigns can potentially reach and impact a broader audience. Looking ahead, future research should delve deeper into the long-term effects of following fitness influencers on health behaviors. There's a clear need to explore targeted, culturally sensitive health promotion efforts that leverage the unique reach and influence of social media. By doing so, we can better understand and utilize this modern medium to foster healthier communities.

## Appendices

Survey on the impact of fitness influencers on health behaviors

*Section One: Demographic and Health Characteristics* Item (Options/Response) Age ( ) years Gender ( ) Male ( ) Female Marital Status ( ) Single ( ) Married Place of Residence (Region) ( ) Southern ( ) Eastern ( ) Northern ( ) Western ( ) Central Educational Level ( ) High School ( ) Bachelor’s Degree ( ) Master’s/Ph.D. Field of Work ( ) Medical ( ) Non-medical ( ) Student ( ) Unemployed Monthly Household Income (SAR) ( ) < 3000 ( ) 3001-10000 ( ) 10001-20000 ( ) > 20000 Weight (in kg): Height (in cm): Are you currently a smoker? ( ) Yes ( ) No Physical activity weekly ( ) None ( ) Once ( ) 2-3 times ( ) > 3 times Do you follow a healthy diet? ( ) Yes ( ) No How do you describe your health status in general: ( ) Poor ( ) Good ( ) Excellent *Section Two: Health-Related Characteristics* Item (Options/Response) Do social media influencers adhere to the health advice they provide to the public? ( ) Always ( ) Sometimes ( ) Never Do you trust health advice given by influencers on social media who are not in the healthcare field? ( ) Always ( ) Sometimes ( ) Never Do you think that the promotion of healthy products by social media influencers is solely for monetary purposes? ( ) Always ( ) Sometimes ( ) Never Have you ever experienced positive results after following the health advice and habits you learned on social media? ( ) No ( ) Yes Have you noticed that influencers who are committed to proper healthy habits tend to have good health? ( ) Some of them ( ) No ( ) Yes Do you agree that social media influencers represent an accurate image of a healthy lifestyle? ( ) Some of them ( ) No ( ) Yes Overall, to what extent are you satisfied with the changes that have occurred in your lifestyle due to the influence of celebrities on social media? ( ) Completely satisfied ( ) Somewhat satisfied ( ) Not satisfied *Section Three: Characteristics of Fitness Influencers* Item (Options/Response) Gender of influencers followed: ( ) Female ( ) Male ( ) Both Average number of influencers followed: ( ) <10 ( ) 10-50 ( ) 50-100 ( ) >100 Approximate average number of followers of influencers followed: ( ) Don’t know ( ) <10k ( ) 10k-100k ( ) 100k-1m ( ) 1m+ Do the influencers on social media whom you specifically choose to follow provide health tips on their platforms? ( ) Yes, all of them ( ) Some of them ( ) Neutral ( ) No Do the influencers on social media whom you follow promote a daily healthy lifestyle? ( ) Yes, all of them ( ) Some of them ( ) Neutral ( ) No
